# Association between haptoglobin and IgM levels and the clinical progression of caseous lymphadenitis in sheep

**DOI:** 10.1186/1746-6148-9-254

**Published:** 2013-12-13

**Authors:** Bruno L Bastos, Dan Loureiro, José T Raynal, Maria T Guedes, Vera Lúcia Costa Vale, Lilia F Moura-Costa, José E Guimarães, Vasco Azevedo, Ricardo W Portela, Roberto Meyer

**Affiliations:** 1 Laboratório de Imunologia e Biologia Molecular, Departamento de Biointeração, Instituto de Ciências da Saúde, Universidade Federal da Bahia, Av. Reitor Miguel Calmon, S/N - Vale do Canela, Salvador, BA CEP 40140-100, Brazil; 2 Laboratório de Análises Clínicas, Departamento de Patologia e Clínicas, Escola de Medicina Veterinária, Universidade Federal da Bahia, Avenida Adhemar de Barros, 500 - Ondina, Salvador, BA CEP 40170-110, Brazil; 3 Laboratório de Genética Molecular e Celular, Departamento de Biologia Geral, Instituto de Ciências Biológicas, Universidade Federal de Minas Gerai, Avenida Antonio Carlos, 6627, Pampulha, Belo Horizonte, MG, Brazil

**Keywords:** Caseous lymphadenitis, Small ruminants, *Quillaja saponaria*, Acute phase response, Haptoglobin, Immunoglobulin M

## Abstract

**Background:**

Sheep caseous lymphadenitis (CLA), caused by *Corynebacterium pseudotuberculosis* (Cp), is associated with direct economic losses and presents significant zoonotic potential. Despite the importance of the disease, a satisfactory vaccine model has not been developed. Thus, this study aimed to investigate the association between haptoglobin (Hp) and IgM levels and the clinical progression of CLA in primarily infected sheep and in sheep immunized with Cp- secreted antigens adjuvanted with *Quillaja saponaria* saponins. These animals were kept with CLA-positive sheep to simulate natural exposure that occurs in field conditions. During the experiment, the Hp and IgM levels were monitored for 21 days, and the development of internal CLA lesions was investigated through necropsies on day182 post-immunization.

**Results:**

Primarily infected sheep in Group 2 (inoculated with 2x10^5^ Cp virulent strain) had higher Hp values between the first and ninth days post inoculation (PI) than sheep in Group 1 (control; P < 0.05). Immunized animals in Group 3 had significantly higher Hp values between the third and seventh days PI, compared with the control group (P < 0.01). Binary logistic regression (BLR) analysis of primarily infected sheep indicated an association between Hp concentration and CLA clinical progression: animals with high Hp values had 99.9% less risk of having CLA abscesses than animals with low Hp levels (Odds ratio = 0.001, P < 0.05). Both experimental groups had significantly higher IgM titers than the control group around the ninth and eleventh days PI (P < 0.05). The BLR analysis for immunized sheep indicated an association between IgM levels and clinical progression: sheep with high IgM titers had 100.0% less risk of having CLA abscesses than animals with low IgM levels (Odds ratio = 0.000, P < 0.05).

**Conclusions:**

Resistance to *C. pseudotuberculosis* infection is supported by the early acute phase response, in which up-regulation of Hp and IgM were predictive of a lower risk of CLA lesion development. Because the immunogen used in this study induced a high production of both Hp and IgM, *Q. saponaria* saponin should be considered a promising candidate in vaccine formulations against sheep CLA.

## Background

Caseous lymphadenitis (CLA) is a chronic infectious disease of sheep and goats caused by the gram-positive bacteria *Corynebacterium pseudotuberculosis* (Cp). In addition to the direct economic losses that may occur due to leather depreciation and weight gain delay [1,2], CLA presents zoonotic potential. Published data strongly indicate that human Cp infection represents an important and significant zoonosis [3].

Despite the importance of the disease, a satisfactory vaccine model for sheep and goats has not been developed. Current knowledge of the immunity induced by Cp indicates that resistance to infection involves both nonspecific and specific host responses. Antibodies help protect against infection [4,5], but for full protection vaccine models must stimulate cellular immunity, such as the activation of CD8+ cells and IFN-γ production by Th1 cells [6-9]. The role of the innate immune system in Cp infection has recently been investigated. It was demonstrated that serum concentrations of haptoglobin (Hp), serum amyloid A and α1 acid glycoprotein were increased in a CLA experimental model in sheep [10]. It was also suggested that sheep that did not develop clinical signs of CLA in field conditions, had significantly higher Hp levels during the acute phase of the disease than sheep that developed superficial abscesses. Although the exact role of Hp in defending against infection by Cp was not identified, data suggested that innate immune mechanisms contributed to the resolution of infection or resistance to the development of CLA pyogranulomas [11].

Considering the hypothesis that full protection against Cp would be achieved by Th1 T cell activation, *Quillaja saponaria*-derived saponins can be considered as potential candidates in vaccine formulations [12]. These saponins have been used in the formulation of veterinary vaccines, stimulating the Th1 response and the production of specific cytolytic T lymphocytes [13]. The aim of this study was to investigate the association between haptoglobin and IgM levels and the clinical progression of CLA (presence or absence of lesions) in primarily infected sheep and in sheep immunized with a saponin-adjuvanted immunogen.

## Methods

### Bacterial strains and secreted antigen production

We used two Cp strains in this study. The T1 strain was used to produce secreted antigens in a previously optimized chemically defined medium (CDM) [14]. This antigenic solution (CDM-secreted antigen) was used as immunogen. A virulent strain of Cp named VD57 was used in the experimental infection model, as previously described [15].

### Animals and experimental inoculations

Fifteen one-year-old Santa Inês ewes were used in the experiment. Santa Inês is a naturalized breed from northeastern Brazil and the experimental animals all came from the same flock. Animal selection was based on several criteria, including body condition, helminth-free state as defined by fecal egg count, normochromic mucosa at visual inspection, normal rectal temperature and absence of abscesses or enlarged lymph nodes on palpation. No sheep had clinical signs of CLA and all were seronegative on an indirect ELISA at the beginning of the study.

We determined our sample size based on the experimental design described previously to investigate the acute phase protein response in an experimental model of ovine caseous lymphadenitis [10]. Factors such as the preliminary (pilot) nature of this study and the requirement of sacrificing all experimental animals to investigate internal lesions in subclinically infected sheep also motivated the use of a small sample size, according to previously published guidelines [16]. Animals were divided into three groups. Group 1 (n = 3) was inoculated with sterile 0.9% saline solution on day zero and kept in an individual pen separated from the flock. Group 2 (n = 6), the experimental infection group, was inoculated with 1 ml of saline containing 2 × 10^5^ CFU of the Cp virulent strain VD57 on day zero. Group 3 (n = 6) was immunized with 1 ml of a solution containing 250 μg of CDM-secreted antigens adjuvanted with 1.5 mg of *Quillaja saponaria* saponin (Sigma-Aldrich) on day zero, and received an immunization booster on day 56. The adjuvant was prepared by dissolving the saponin at a concentration of 150 mg/ml in sterile 0.9% saline solution as a stock solution and filtering through a 0.22 μm membrane. On the immunization days, the saponin stock solution was added to the secreted antigen to a final concentration of 1.5 mg/ml [17], and the mixture was stirred for 30 minutes before administration. All inoculations were performed subcutaneously in the right flank. An additional 12 CLA-positive sheep, presenting external lesions and positive serology by ELISA, were kept with Groups 2 and 3 from day zero (time of inoculation/immunization) until day 182 (last day of observation) to simulate natural exposure that occurs in field conditions. These animals were kept on pasture during the day, with free access to water, and returned to pens by the end of each day to receive protein and mineral supplementation.

This experiment was conducted under the authority of Brazilian Law nº11.794, 8 October 2008 (statements on the use of animals in research experiments), after approval by the Secretariat of Animal Health of the Ministry of Agriculture, Livestock and Food Supply. Slaughter was conducted at a slaughterhouse supervised by the National Meat Inspection Service of the same Ministry.

### Sample collection and analysis

Blood samples were collected before inoculation/immunization (day zero) and on days 1, 3, 5, 7, 9, 11, 14 and 21 post-inoculation (PI). On each occasion, one blood aliquot was collected using vacuum tubes without anticoagulant and allowed to clot at room temperature, for serum collection. These samples were tested to determine Hp and IgM levels.

Serum Hp concentration was estimated based on hemoglobin-binding capacity in microtitration plates, as previously described [11]. A standard curve was developed with standard Hp solutions (Sigma-Aldrich) diluted from 1.050 to 0.008 g/L. Fifty μL of Hp standard or serum sample was added to 50 μL of 0.9% saline solution in each well. Next, 50 μL of sheep methemoglobin solution (30 mg/dL) was added and plates were incubated for 10 minutes at 20°C. A blank (50 μL of 0.9% saline) was run with each serum sample. Following incubation, a guaiacol reagent (150 μL, pH 4.0) and 50 μL of H_2_O_2_ solution (0.02 mol/L) were added. After 5 minutes, absorbance at 490 nm was measured using a microplate reader (Bio-Rad, Hercules, CA).

The indirect ELISA for detection of Cp*-*specific IgM antibodies was performed as previously described [11]. ELISA microplates were sensitized with Cp supernatant antigens (1:100 in carbonate-bicarbonate buffer, pH 9.6) and incubated at 4°C for 16 hours. The blocking step was made with 5% skim milk in PBS-T and incubated for 2 hours at 37°C. Serum samples were pre-treated (v/v 1:1) with Dynabeads Protein G (Invitrogen, Carlsbad, CA), and 100 μL of these samples were added to the plates in duplicate and incubated for 1 hour at 37°C. A rabbit anti-ovine IgM polyclonal antibody conjugated with horseradish peroxidase (Serotec, Raleigh, NC) was used as detection antibody (1:20,000 in PBS-T) and incubated for 45 minutes at 37°C. Finally, the reaction was developed with 100 μL/well of a solution containing H_2_O_2_ and tetramethylbenzidine (Moss Inc., Pasadena, MD) for 15 minutes, and stopped with 4 N H_2_SO_4_. Results were read in an ELISA plate reader (Bio-Rad) at 450 nm.

Although blood sampling focused on the acute phase period (until the 21^st^ day), clinical assessment continued for six months, with all animals clinically examined twice per month through the inspection of superficial lymph nodes and routine blood sampling. External abscesses found during the experiment period were sampled through an incision with sterile surgical equipment, followed by drainage of purulent material into sterile flasks for bacteriological assays. After the 182-day observation period, animals were necropsied at the Baby Bode slaughterhouse (Feira de Santana, BA, Brazil), which has an official meat inspection system and performs postmortem pathology inspections. All external and internal lymph nodes, and tissue samples with lesions suspected to be caused by Cp, were collected and microbiologically assayed according to previously described protocols [15,18].

### Statistical analysis

Data were analyzed using IBM SPSS statistics, version 20.0. The distribution of Hp and IgM results was graphically expressed through boxplots for each sampling time. Results were compared between groups, and significant differences (P < 0.05) were determined using the nonparametric Mann–Whitney test. Based on the summary of records of external abscesses and post-mortem examination (Table 1), a second series of graphics was made using the animals’ data clustered by their CLA clinical status: with CLA lesions (n = 5 in Group 2, n = 3 in Group 3) or without CLA lesions (n = 1 in Group 2, n = 3 in Group 3). Binary logistic regression analysis was used to examine the association between variables in Groups 2 and 3 through the odds ratio (OR) calculation [1,19]. The presence or absence of CLA lesions was treated as the predicted dichotomous categorical variable (outcome), while Hp and IgM levels during the acute phase were considered as predictor continuous variables. A model construct was done using a forward selection of the variables. Adequacy of the final models was assessed by the Hosmer and Lemeshow goodness-of-fit test (P). P values < 0.05 were considered significant. Complementary tables presenting the single haptoglobin (g/l) and IgM (optical density at 490 nm) values for each sheep during the acute phase period are provided as additional files.

**Table 1:** **Summary of external abscess records and *post-mortem *** **examination results**

**Group**	**Animals**	**Post mortem examination**	**Final diagnosis**
#1 - Control animals inoculated with sterile 0.9% saline solution	# 001	No abscess	**----**
	# 002	No abscess	**----**
	# 003	No abscess	**----**
# 2 - Experimental infection with 2 × 10^5^ CFU *C. pseudotuberculosis* VD57 wild strain	# 122	Three abscesses in the inoculation site, one in the right cervical lymph node and 17 abscesses in the lungs	Positive
	# 150	Eight abscesses in the lungs	Positive
	# 156	One abscess in the lungs and one in the liver	Positive
	# 181	Two abscesses in the lungs	Positive
	# 189	One abscess in the lungs and one in the liver	Positive
	# 602	No abscess	Negative
# 3 - Immunization with 250 μg CDM antigen and 1.5 mg saponins	# 51	One abscess in the left parotid lymph node and one in the liver	Positive
	# 61	One abscess in the left cervical lymph node	Positive
	# 141	One abscess in the right parotid lymph node	Positive
	# 155	No abscess	Negative
	# 161	No abscess	Negative
	# 295	No abscess	Negative

The final diagnosis was used to cluster animals by their disease status: with CLA lesions (positive) or without CLA lesions (negative).

## Results

The results of CLA lesion detection are presented in Table 1. Group 1 (control) animals did not develop any lesions. In Groups 2 and 3, there was a notable absence of clinical signs of infection or inflammatory response during the 182 days post-infection, except for one animal (#122) in Group 2, which developed an abscess at the inoculation site. At slaughter, internal abscesses were recorded in different sites and organs in five sheep in Group 2 (animals # 122, 150, 156, 181 and 189) and in three sheep in Group 3 (animals # 51, 61 and 141). The number of lesions in each animal ranged from one to 18 in the lungs, liver and lymph nodes. All purulent content of lesions had positive Cp isolation and identification. A particular tropism for the lungs was detected in the primarily infected animals (Group 2), but not in the immunized sheep that presented CLA lesions. One sheep from Group 2 (#602) was naturally resistant to Cp infection and did not develop any pyogranulomas or CLA abscesses. Three sheep in Group 3 presented no signs of CLA (animals # 155, 161 and 295).

**Figure 1:**
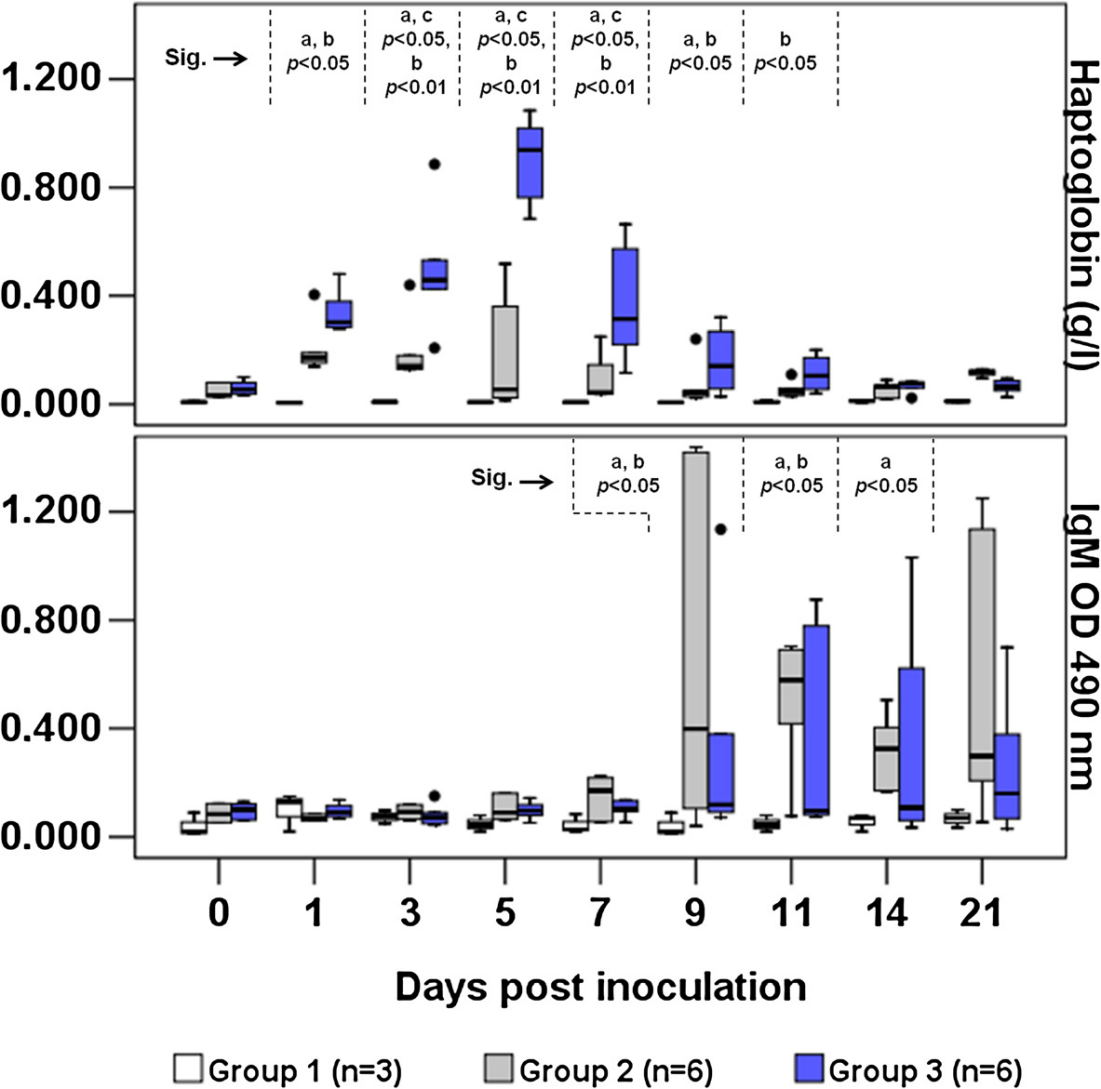
**Distribution of haptoglobin and IgM values in sheep during CLA’s acute phase period.** Values are expressed as boxplot graphics, in which boxes represent the median value (line within box) and 25^th^ and 75^th^ percentiles (bottom and top of box); whiskers represent the 2.5^th^ and 97.5^th^ percentiles; and dots represent outliers. Group 1, control group: animals inoculated with 0.09% saline solution; Group 2: inoculation with 2 × 10^5^ CFU of *Corynebacterium* pseudotuberculosis (Cp) VD57 wild strain; Group 3: immunization with 250 μg of CDM-secreted antigen plus 1.5 mg saponin. Significant differences (Sig.) between groups in Mann–Whitney test are presented at the top of each graphic with superscript letters: a = Groups 1 and 2, b = Groups 1 and 3, c = Groups 2 and 3.

The distribution of Hp values for each group during the acute phase is shown in Figure 1. The primarily infected sheep in Group 2 presented a typical acute phase response curve with significantly higher (P < 0.05) Hp values occurring between days 1 and 9 PI, in comparison to Group 1 (control). The immunized animals in Group 3 showed a strong acute phase response, with Hp values significantly higher (P < 0.05) than those of the control group between days 1 and 11 PI. The highest Hp values were detected between the third and seventh days PI in Group 3, a notably significant difference in relation to Group 1 (P < 0.01). Sheep in Group 3 had higher Hp values than Group 2 animals on days 3, 5 and 7 PI (P < 0.05). Both groups’ Hp levels had returned to baseline by day 14 PI.

**Figure 2:**
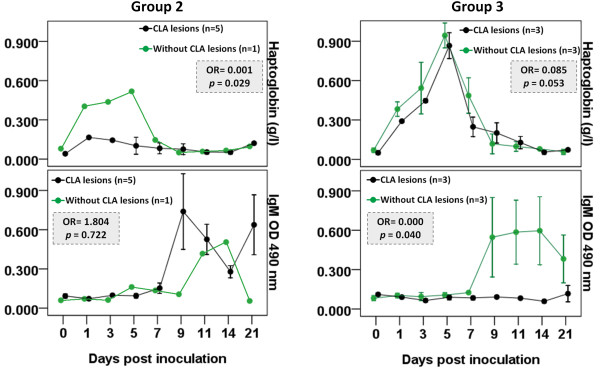
**Haptoglobin and IgM values in Groups 2 and 3 according to CLA status.** Values are expressed as mean ± standard error. Sheep were clustered by the absence (green) and presence (black) of CLA lesions. Group 2: inoculation with 2 × 10^5^ CFU of Cp VD57 wild strain; Group 3: immunization with 250 μg of CDM-secreted antigen plus 1.5 mg saponin. The association between variables and the clinical progression (presence or absence of CLA lesions) was examined through binary logistic regression, and odds ratios (OR) are presented in grey boxes. Association was considered significant if the P value was less than 0.05. The Hosmer and Lemeshow test P values were 0.221 (Group 2) and 0.803 (Group 3).

Hp values clustered according to the presence or absence of CLA lesions are presented in Figure 2. The highest measured Hp value (0.518 g/l), found in sheep #602 in Group 2, was 3.1 times higher than the highest mean Hp value (0.166 g/l) in other animals from the same group. Sheep #602 was the animal naturally resistant to Cp infection with no CLA lesion development. (See Additional file 1 for single Hp values.) Binary logistic regression analysis indicated a significant association between high Hp values and the absence of CLA lesions (P < 0.05; OR = 0.001).

The distribution of IgM values for each group is presented in Figure 1. (See Additional file 2 for single IgM values.) Experimental infection with 2 × 10^5^ CFU of Cp virulent strain VD57 induced higher IgM responses in Group 2 than immunization did in Group 3, but these differences were not significant. Both experimental groups had significantly higher IgM values on days 9 and 11 PI compared with the control group (P < 0.05). These results suggest that the adaptive immune response can first be measured on day 9 PI.

IgM data clustered according to the presence or absence of CLA lesions are presented in Figure 2. No association with outcomes was detected in Group 2. All sheep experimentally infected with 2 × 10^5^ CFU Cp virulent strain had elevated IgM levels during the acute phase period. However, in Group 3, only those animals that did not develop CLA abscesses had high IgM titers between days 9 and 21 PI. Binary logistic regression analysis indicated a significant association between high IgM values and the absence of CLA lesions (P < 0.05; OR = 0.000).

## Discussion

CLA is an important disease that affects sheep and goats, with no satisfactory commercially available vaccine. In developing a reliable immunogen it is important to consider the acute immune response, and IgM and Hp are among the most important parameters during this phase of the disease. Our aim in this study was to observe the kinetics of these two acute phase markers in the progression of CLA lesion development.

Three sheep in Group 3 (animals # 51, 61 and 141) developed CLA abscesses, probably as a result of natural exposure to sheep with clinical CLA and draining lesions. The observed tropism for the lungs was previously described in a study evaluating lesions in mice inoculated with four equine Cp strains, which demonstrated distinct tropism for the liver, spleen, lungs or mesenteric lymph nodes [20]. In this study, because the animals were naturally exposed to the pathogen, it is very difficult to define the tropism of the circulating strain in the flock.

The saponin-adjuvanted CDM-secreted antigen induced a strong acute phase response between days 1 and 7 after immunogen inoculation. Haptoglobin is one of the most sensitive acute phase markers of inflammatory and infectious conditions in ruminants. This protein can be used to assess the innate immune response to vaccination in sheep [21]. The existence of an Hp reaction in ovine CLA has been previously reported, occurring approximately 7 days PI [10]. However, our data show that this acute phase response can be detected from day 1 PI, as shown in Figure 1.

The OR obtained for Group 2 shows that the animal with high Hp values during the acute phase response had 99.9% less risk of developing CLA abscesses than animals with low Hp levels. Although Group 3 had the highest Hp levels, no association between Hp levels and CLA lesion development could be detected. We were unable to detect an association because inoculation with the saponin-adjuvanted immunogen induced a strong Hp reaction in all sheep. It has been suggested that a high Hp level during the acute phase of Cp infection represents a good prognosis for the clinical progression of CLA in sheep [11]. One possible role of Hp against infection is related to iron metabolism: high levels of serum Hp might contribute to iron sequestration, making the mineral less available for bacterial growth, as in the case of *Mycobacterium tuberculosis* infection [22].

The OR calculated for Group 3 indicated that sheep with high IgM titers during the acute phase period had 100.0% less risk of developing CLA abscesses than animals with low IgM levels. Few studies regarding IgM antibody levels during Cp infection have been published to date, but our results agree with studies demonstrating the protective effect of the humoral antigen-specific IgM response against *Nocardia brasiliensis*[23,24] and *Mycobacterium tuberculosis*[25].

Based on the Hp and IgM results of Group 3, it is possible to infer that the saponin-adjuvanted CDM-secreted antigen induced early defense mechanisms during the acute phase period. However, it is not plausible to consider Hp and IgM as uniquely responsible for protection against CLA. Although few vaccine models using saponins as adjuvant have been tested in veterinary medicine, prior studies have shown promising results in protecting against murine visceral leishmaniasis [26], bovine respiratory syncytial virus [27] and swine foot-and-mouth disease virus [28]. These results indicate that saponins may be a good choice for adjuvant in CLA vaccine models.

## Conclusions

Establishing the kinetics of Hp and IgM production during the acute phase of infection in our two models enabled us to characterize the association of these variables with the clinical progression of CLA in sheep. The results indirectly demonstrate that resistance to Cp infection is supported by the early acute phase response, with up-regulation of Hp and IgM predicting a lower risk of developing CLA lesions. Because the immunogen used in this study induced high production of both Hp and IgM, *Quillaja saponaria* saponin should be considered as a promising candidate in CLA vaccine formulations. Understanding the pathways initially activated by Cp antigens is crucial to construct an effective vaccine model. We highly recommend further studies on the modulation of immunity by adjuvants during the acute phase of CLA using a wider variety of immunological markers.

## Abbreviations

Cp: *Corynebacterium pseudotuberculosis*; CLA: Caseous lymphadenitis; PI: Post-inoculation; Hp: Haptoglobin; IgM: Immunoglobulin M; BHI: Brain heart infusion; PBS-T: Phosphate-buffered saline with 0.05% Tween 20; BLR: Binary logistic regression; OR: Odds ratio.

## Competing interests

All authors have no competing interests.

## Authors’ contributions

BLB undertook sample collection and laboratory experiments, analyzed the results and drafted the manuscript. DL, JTR and MTG were responsible for laboratory experiments. JEG and VA contributed to the design of field data collection. VLCV, LFMC, RWP and RM contributed to the design, writing of the manuscript and coordination of the study. All authors have read and approved the manuscript.

## Supplementary Material

Additional file 1 Complementary table presenting the single haptoglobin value (g/l) for each sheep during the acute phase period. Click here for file

Additional file 2 Complementary table presenting the single IgM value (optical density at 490 nm) for each sheep during the acute phase period. Click here for file
