# Supplementary material for: Association between haptoglobin and IgM levels and the clinical progression of caseous lymphadenitis in sheep
Source: BMC Vet Res. 2013 Dec 13;9:254. doi: 10.1186/1746-6148-9-254 (PMC3866939; doi:10.1186/1746-6148-9-254)
Supplement: Additional file 1 — Complementary table presenting the single haptoglobin value (g/l) for each sheep during the acute phase period. [file 1746-6148-9-254-S1.pdf]

Additional file 1

Complementary table presenting the single haptoglobin value (g/l) for each sheep along the acute phase period.

Group	Animals	Days post inoculation								
		0	1	3	5	7	9	11	14	21
# 1	# 001	0,012	0,006	0,010	0,008	0,008	0,006	0,007	0,013	0,011
	# 002	0,009	0,007	0,009	0,010	0,009	0,008	0,014	0,006	0,012
	# 003	0,005	0,005	0,004	0,005	0,003	0,007	0,007	0,013	0,009
#2	# 122	0,080	0,140	0,180	0,360	0,250	0,240	0,110	0,090	0,110
	# 150	0,034	0,154	0,140	0,060	0,044	0,023	0,044	0,064	0,125
	# 156	0,028	0,190	0,130	0,014	0,037	0,046	0,034	0,022	0,123
	# 181	0,028	0,178	0,142	0,024	0,042	0,041	0,029	0,017	0,118
	# 189	0,034	0,166	0,128	0,050	0,039	0,028	0,049	0,069	0,130
	# 602	0,080	0,403	0,437	0,518	0,146	0,050	0,058	0,066	0,097
# 3	# 141	0,080	0,313	0,441	0,684	0,116	0,057	0,040	0,023	0,060
	# 155	0,070	0,290	0,531	1,084	0,664	0,268	0,172	0,076	0,027
	# 161	0,100	0,480	0,208	0,984	0,221	0,057	0,068	0,079	0,096
	# 295	0,040	0,379	0,889	0,763	0,573	0,028	0,056	0,083	0,050
	# 51	0,039	0,283	0,424	0,894	0,372	0,320	0,201	0,081	0,070
	# 61	0,031	0,277	0,474	1,020	0,255	0,226	0,144	0,061	0,087
