# Supplementary material for: Association between haptoglobin and IgM levels and the clinical progression of caseous lymphadenitis in sheep
Source: BMC Vet Res. 2013 Dec 13;9:254. doi: 10.1186/1746-6148-9-254 (PMC3866939; doi:10.1186/1746-6148-9-254)
Supplement: Additional file 2 — Complementary table presenting the single IgM value (optical density at 490 nm) for each sheep during the acute phase period. [file 1746-6148-9-254-S2.pdf]

Additional file 2

Complementary table presenting the single IgM value (optical density at 490 nm) for each sheep along the acute phase period.

Group	Animals	Days post inoculation								
		0	1	3	5	7	9	11	14	21
# 1	# 001	0,090	0,020	0,050	0,080	0,085	0,090	0,080	0,070	0,100
	# 002	0,010	0,150	0,098	0,045	0,028	0,010	0,045	0,080	0,070
	# 003	0,020	0,130	0,075	0,020	0,020	0,020	0,020	0,020	0,035
#2	# 122	0,110	0,069	0,116	0,162	0,208	0,041	0,078	0,403	0,385
	# 150	0,053	0,086	0,121	0,092	0,054	1,439	0,691	0,171	1,249
	# 156	0,123	0,059	0,065	0,065	0,219	0,401	0,583	0,321	0,210
	# 181	0,123	0,060	0,069	0,064	0,225	0,395	0,574	0,329	0,208
	# 189	0,053	0,085	0,119	0,088	0,057	1,419	0,704	0,168	1,135
	# 602	0,060	0,071	0,062	0,162	0,135	0,106	0,417	0,505	0,055
# 3	# 141	0,118	0,087	0,055	0,099	0,106	0,112	0,091	0,081	0,084
	# 155	0,063	0,076	0,089	0,080	0,105	0,127	0,102	0,135	0,068
	# 161	0,122	0,137	0,151	0,144	0,137	1,135	0,876	0,622	0,378
	# 295	0,062	0,096	0,044	0,095	0,134	0,379	0,779	1,032	0,699
	# 51	0,085	0,068	0,050	0,052	0,054	0,092	0,083	0,035	0,238
	# 61	0,131	0,116	0,090	0,119	0,094	0,072	0,074	0,061	0,030
